# Supplementary material for: Combining bacteriophage and vancomycin is efficacious against MRSA biofilm-like aggregates formed in synovial fluid
Source: Front Med (Lausanne). 2023 Jun 9;10:1134912. doi: 10.3389/fmed.2023.1134912 (PMC10289194; doi:10.3389/fmed.2023.1134912)
Supplement: Supplementary file 1 [file Table_1.docx]

**Supporting Information**

**Table S1.** *Galleria mellonella* health index scoring system.

| **Category** | **Observation** | **Score** |
| --- | --- | --- |
| Activity | No activity | 0 |
|  | Minimal response to stimuli | 1 |
|  | Active response to stimuli | 2 |
|  | Active without stimulation | 3 |
