# Supplementary material for: Combining bacteriophage and vancomycin is efficacious against MRSA biofilm-like aggregates formed in synovial fluid
Source: Front Med (Lausanne). 2023 Jun 9;10:1134912. doi: 10.3389/fmed.2023.1134912 (PMC10289194; doi:10.3389/fmed.2023.1134912)
Supplement: Supplementary file 2 [file Table_2.docx]

**Supporting Information**

**Table S2. InterProScan functional domain results for phage Remus**

| **Gene product; protein ID** | **Name** | **Type** | **Minimum (AA)** | **Maximum (AA)** | **Length (AA)** |
| --- | --- | --- | --- | --- | --- |
| 134; YP_008431175.1 | Coil | COILS | 96 | 116 | 21 |
|  | DUF2977 | Pfam | 14 | 74 | 61 |
|  | hypothetical protein CDS | CDS | 1 | 119 | 119 |
| 137; YP_008431172.1 | DUF4815 | Pfam | 9 | 611 | 603 |
|  | DUF4815 domain-containing protein CDS | CDS | 1 | 1153 | 1153 |
| 138; YP_008431171.1 | structural protein CDS | CDS | 1 | 174 | 174 |
|  | baseplate structural protein gp8, domain 1 | Gene3D | 6 | 106 | 101 |
| 140; YP_008431169.1 | baseplate J/gp47 family protein CDS | CDS | 1 | 349 | 349 |
|  | Baseplate_J | Pfam | 66 | 308 | 243 |
| 141; YP_008431168.1 | baseplate protein CDS | CDS | 1 | 235 | 235 |
|  | LysM | CDD | 6 | 41 | 36 |
|  | LysM_2 | SMART | 5 | 66 | 62 |
|  | gpW/gp25-like | Superfamily | 125 | 212 | 88 |
|  |  | Gene3D | 110 | 221 | 112 |
|  | LysM domain | Gene3D | 3 | 76 | 74 |
| 144; YP_008431165.1 | glycerophosphoryl diester phosphodiesterase CDS | CDS | 1 | 819 | 819 |
|  | Phosphatidylinositol (PI) phosphodiesterase | Gene3D | 601 | 818 | 218 |
|  | GP_PDE | PROSITE_PROFILES | 593 | 818 | 226 |
|  | GLYCEROPHOSPHORYL DIESTER PHOSPHODIESTERASE | Panther | 603 | 817 | 215 |
|  | GDPD | Pfam | 606 | 812 | 207 |
|  | PLC-like phosphodiesterases | Superfamily | 618 | 816 | 199 |
|  | Coil | COILS | 406 | 426 | 21 |
|  | Coil | COILS | 349 | 369 | 21 |
|  | Coil | COILS | 316 | 336 | 21 |
| 145; YP_008431165.1 | endopeptidase domain like (3.90.1720.10) | Gene3D | 151 | 293 | 143 |
|  | NLPC_P60 | PROSITE_PROFILES | 146 | 290 | 145 |
|  | NLPC_P60 | Pfam | 164 | 259 | 96 |
|  | Cysteine proteinases | Superfamily | 156 | 289 | 134 |
|  | tail lysin CDS | CDS | 1 | 297 | 297 |
| 146; YP_008431163.1 | endopeptidase domain like (3.90.1720.10) | Gene3D | 688 | 807 | 120 |
|  | CHAP | PROSITE_PROFILES | 676 | 807 | 132 |
|  | CHAP | Pfam | 695 | 781 | 87 |
|  | Cysteine proteinases | Superfamily | 687 | 795 | 109 |
|  | disorder_prediction | MOBIDB_LITE | 645 | 670 | 26 |
|  | disorder_prediction | MOBIDB_LITE | 645 | 662 | 18 |
|  | CHAP domain-containing protein CDS | CDS | 1 | 811 | 811 |
| 147; YP_008431162.1 | hypothetical protein CDS | CDS | 1 | 277 | 277 |
|  | Coil | COILS | 18 | 38 | 21 |
|  | 1.10.530.10 | Gene3D | 52 | 196 | 145 |
|  | Phage_lysozyme2 | Pfam | 61 | 196 | 136 |
|  | disorder_prediction | MOBIDB_LITE | 35 | 59 | 25 |
|  | disorder_prediction | MOBIDB_LITE | 1 | 20 | 20 |
|  | disorder_prediction | MOBIDB_LITE | 1 | 15 | 15 |
| 148; YP_008431161.1 | 1.10.530.10 | Gene3D | 115 | 231 | 117 |
|  | Glucosaminidase | Pfam | 120 | 229 | 110 |
|  | Lysozyme_4 | SMART | 106 | 242 | 137 |
|  | disorder_prediction | MOBIDB_LITE | 18 | 40 | 23 |
|  | disorder_prediction | MOBIDB_LITE | 18 | 37 | 20 |
|  | tail tip protein CDS | CDS | 1 | 290 | 290 |
