# Supplementary figures and images for: Combining bacteriophage and vancomycin is efficacious against MRSA biofilm-like aggregates formed in synovial fluid
Source: Front Med (Lausanne). 2023 Jun 9;10:1134912. doi: 10.3389/fmed.2023.1134912 (PMC10289194; doi:10.3389/fmed.2023.1134912)

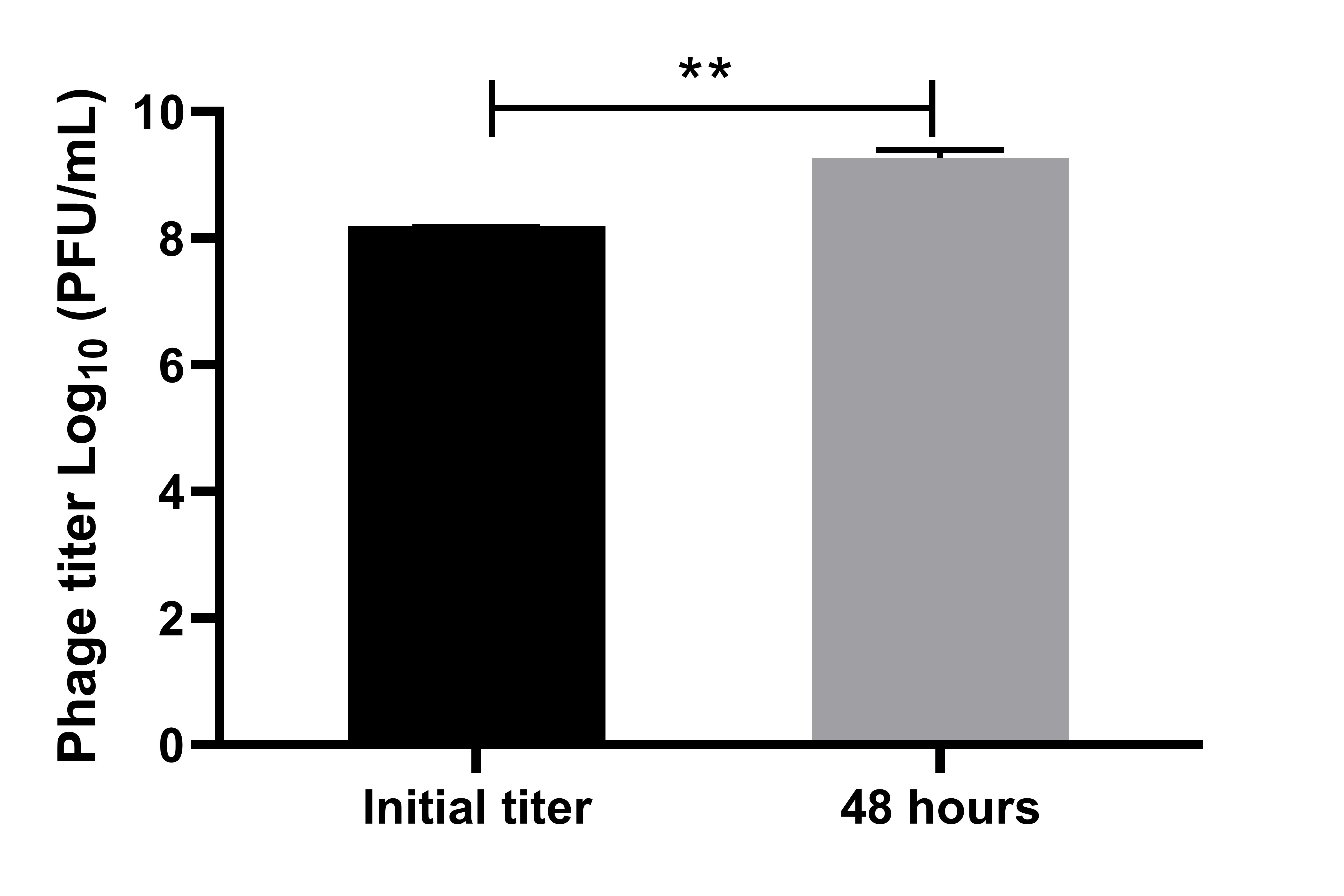

Supplement: Supplementary Figure 1 — Phage Remus titer in synovial fluid-derived aggregates of Staphylococcus aureus BP043 aggregates. Remus density was checked after 48 h at 37°C and compared to initial titer. N = 4, ± SE. Statistical significance was performed using t-test (two-tailed, unpaired). **p < 0.01. [file Image_1.JPEG]

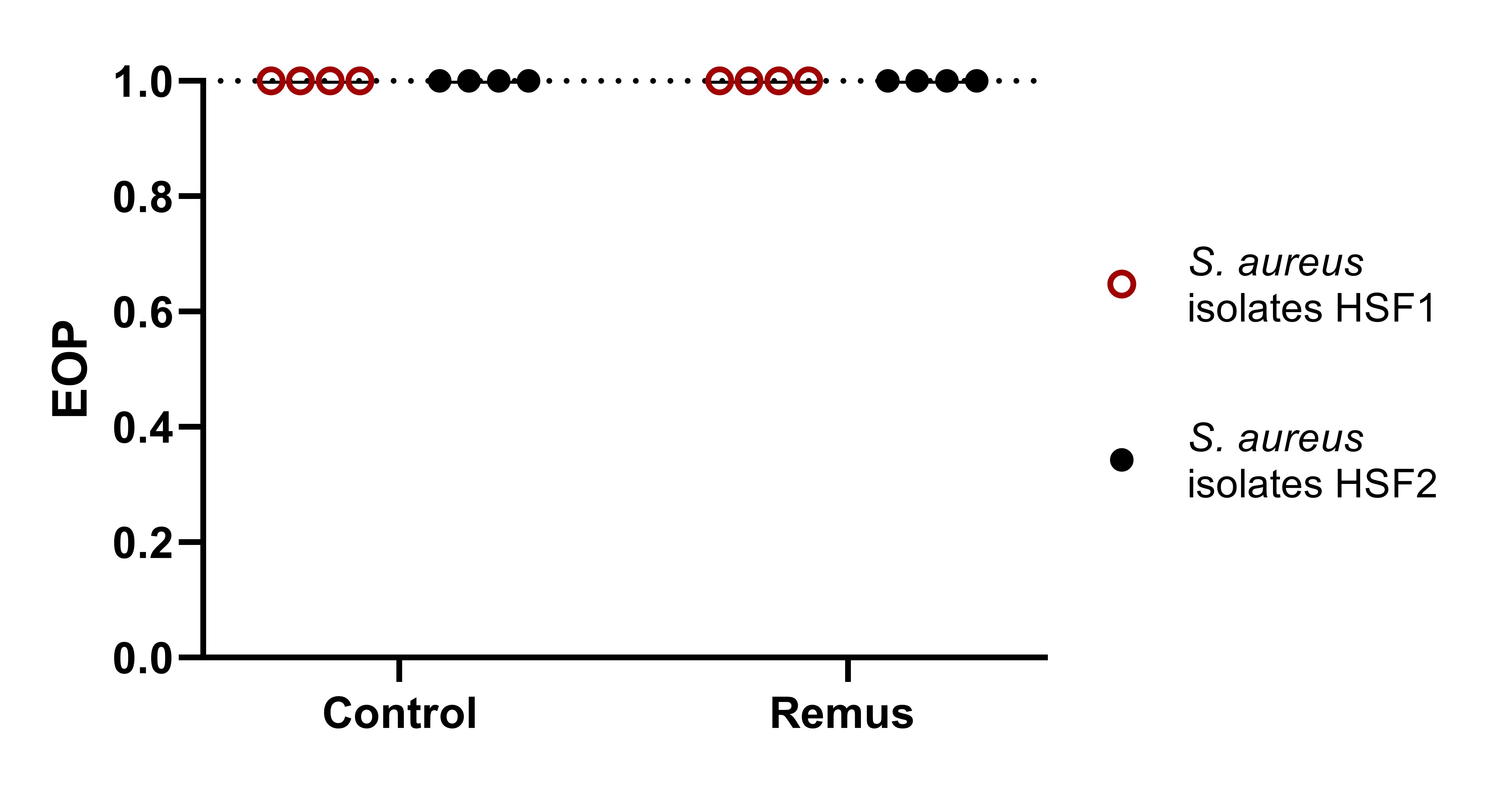

Supplement: Supplementary Figure 2 — No development of resistance by Staphylococcus aureus BP043 against Remus. The rise of phage-resistance sub-population was monitored by checking the efficiency of plating (EOP) of the S. aureus BP043 isolates that survived the 48 h Remus treatment or for the control (no Remus treatment) in synovial fluid (SF). EOP was calculated by dividing Remus titer on the tested S. aureus BP043 by Remus titer on the ancestor S. aureus BP043. Two human synovial fluids were used (HSF1, HSF2), and four S. aureus isolates were checked for resistance per synovia fluid. [file Image_2.jpg]
